# Management of intoxicated patients – a descriptive outcome analysis of 4,267 ICU patients

**DOI:** 10.1186/s12873-022-00602-y

**Published:** 2022-03-12

**Authors:** Richard Rezar, Christian Jung, Behrooz Mamandipoor, Clemens Seelmaier, Thomas K. Felder, Michael Lichtenauer, Sarah Wernly, Samanta M. Zwaag, Dylan W. De Lange, Bernhard Wernly, Venet Osmani

**Affiliations:** 1 Department of Cardiology and Intensive Care Medicine, Paracelsus Medical University of Salzburg, Müllner Hauptstraße 48, 5020 Salzburg, Austria; 2 Department of Cardiology, Pulmonology and Vascular Medicine, Medical Faculty, Heinrich-Heine-University Düsseldorf, Moorenstraße 5, 40225 Duesseldorf, Germany; 3 Fondazione Bruno Kessler Research Institute, Trento, Via Sommarive 18 - Povo, 38123 Trento, Italy; 4 Department of Laboratory Medicine, Paracelsus Medical University of Salzburg, Müllner Hauptstraße 48, 5020 Salzburg, Austria; 5 Department of Internal Medicine, General Hospital Oberndorf, Paracelsusstraße 37, 5110 Oberndorf, Austria; 6 Department of Intensive Care Medicine, University Medical Center, University Utrecht, Heidelberglaan 100, 3584 CX Utrecht, The Netherlands; 7 Department of Anaesthesiology, Perioperative Medicine and Intensive Care Medicine, Paracelsus Medical University of Salzburg, Müllner Hauptstraße 48, 5020 Salzburg, Austria; 8 Center for Public Health and Healthcare Research, Paracelsus Medical University of Salzburg, Strubergasse 21, 5020 Salzburg, Austria

**Keywords:** Epidemiology, Poisoning, Hospital mortality, Intensive care units, Critical illness

## Abstract

**Introduction:**

Intoxications are common in intensive care units (ICUs). The number of causative substances is large, mortality usually low. This retrospective cohort study aims to characterize differences of intoxicated compared to general ICU patients, point out variations according to causative agents, as well as to highlight differences between survivors and non-survivors among intoxicated individuals in a large-scale multi-center analysis.

**Methods:**

A total of 105,998 general ICU patients and 4,267 individuals with the admission diagnoses “overdose” and “drug toxicity” from the years 2014 and 2015 where included from the eICU Collaborative Research Database. In addition to comparing these groups with respect to baseline characteristics, intensive care measures and outcome parameters, differences between survivors and non-survivors from the intoxication group, as well as the individual groups of causative substances were investigated.

**Results:**

Intoxicated patients were younger (median 41 vs. 66 years; *p*<0.001), more often female (55 vs. 45%; *p*<0.001), and normal weighted (36% vs. 30%; *p*<0.001), whereas more obese individuals where observed in the other group (37 vs. 31%; *p*<0.001). Intoxicated individuals had a significantly lower mortality compared to general ICU patients (1% vs. 10%; aOR 0.07 95%CI 0.05-0.11; *p*<0.001), a finding which persisted after multivariable adjustment (aOR 0.17 95%CI 0.12-0.24; *p*<0.001) and persisted in all subgroups. Markers of disease severity (SOFA-score: 3 (1-5) vs. 4 (2-6) pts.; *p*<0.001) and frequency of vasopressor use (5 vs. 15%; *p*<0.001) where lower, whereas rates of mechanical ventilation where higher (24 vs. 26%; *p*<0.001) in intoxicated individuals. There were no differences with regard to renal replacement therapy in the first three days (3 vs. 4%; *p*=0.26). In sensitivity analysis (interactions for age, sex, ethnicity, hospital category, maximum initial lactate, mechanical ventilation, and vasopressor use), a trend towards lower mortality in intoxicated patients persisted in all subgroups.

**Conclusion:**

This large-scale retrospective analysis indicates a significantly lower mortality of intoxicated individuals compared to general ICU patients.

## Introduction

Intoxications are common, hence responsible for a large number of emergency ward presentations and subsequent intensive care admissions [1]. Despite the large quantity of intoxicated individuals, the actual count of patients requiring monitoring is limited and the number of severe courses and deaths is comparatively low [2, 3]. As per the American Association of Poison Control Center´s annual report, 2,148,141 exposures to poison in humans were observed in the United States (US) in 2019, whereas only in 40,058 cases (1.8%) a major effect by the toxin on an individual’s outcome was observed. In contrast, 1,923 deaths (0.09%) due to poisoning were registered. In total, 658,242 poisoned patients were treated in US health-care facilities, of which 96,483 (14.6%) were admitted to an intensive care unit (ICU) [4]. According to the Global Burden of Disease Study, 86,400 deaths worldwide (0.15% of all deaths) were caused by poisoning in 2015 [5].

Overall, there are many studies on poisoning in the medical literature, but only few involve large case numbers. Two of the largest papers in the field from the years 2008 to 2011 deal with European patient collectives and show a comparatively low in-hospital mortality in this very often heterogeneous group of patients (1-2%) [1, 2]. Furthermore, there are many studies on various specific toxins or local investigations of individual intensive care units examining distinct characteristics of causing substances and/or affected patients. The topics of these studies range from large multi-center studies, national health care cost analyses, biomarker validation studies to highly specific topics such as workup of Scorpion envenomation cases [6–8]. With this study, we sought not only to examine a large U.S. intensive care database in terms of outcome, but also to compare the outcome of the subgroup of intoxicated patients with the rest of all intensive care individuals in the same collective at the same time. Furthermore, we also tried to contrast not only epidemiological characteristics but also patient-care specific data of the two groups, and then compare in detail survivors and non-survivors of intoxicated patients, as well as the groups of each causative agent based on the same characteristics, to get a better picture of this heterogeneous diagnostic group.

The well-known problem is that only a small proportion of intoxicated patients are truly critically ill or at least require monitoring. Considering the limited availability and high costs of ICU beds, it is important to perform resource allocation appropriately without endangering human lives [9].

Several steps are necessary to counteract these problems. First, the population of intoxicated patients in intensive care units must be accurately characterized and their outcome analyzed. Further, it is necessary to develop prediction models in large cohorts that ideally filter out all patients who are likely to suffer from a complicated clinical course. Subsequently, these models have to be tested prospectively and possible pitfalls have to be learned from their application in practice. Finally, the models can be incorporated into routine procedures. In our analysis from multiple U.S. intensive care units, we seek to contribute important data to the first step. We provide an overview of a large cohort of general ICU patients (*n*=105,998) in the database compared with all intoxicated patients (*n*=4,267). Thereafter, we present a detailed analysis regarding survivors and non-survivors, as well as differences with regard to individual substance categories.

## Methods

### Study Subjects

Critically ill patients admitted to an ICU were included in this analysis from the multi-centre eICU Collaborative Research Database [10]. In accordance with national legislation and institutional requirements, written informed consent was not required for participation as this is an analysis of a publicly available, deidentified database with pre-existing institutional review board (IRB). An Institutional Review Board (IRB; Massachusetts Institute of Technology, Cambridge, MA, USA) approval was obtained for the creation of the database. The present study is an analysis of the publicly available anonymized database; therefore no further ethics approval was deemed necessary. The eICU database includes admissions of 335 ICUs across the USA in 2014 and 2015 and is released under the Health Insurance Portability and Accountability Act (HIPAA) safe harbor provision [10, 11]. Patients were classified by trained eICU clinicians according to Acute Physiology And Chronic Health Evaluation (APACHE) IV model [10]. Only unique admissions of adult patients with available APACHE IV but no readmissions were included in the final analysis. In total 110,265 patients from the eICU database were included in the overall analysis, whereas intoxicated patients (*n*=4,267; diagnosis categories “drug toxicity” and sub-categories of "overdose") were looked at in detail.

### Statistical analysis

Baseline characteristics were extracted, continuous data expressed as median ± interquartile range, and differences between independent groups calculated accordingly using Kruskal-Wallis equality rank test. Categorical data are expressed as numbers (percentages), and the chi-square test was used to calculate univariate differences between groups. In addition to standard laboratory parameters, relevant intensive care measures (mechanical ventilation; use of vasopressors; dialysis in the first three days) were extracted from the database. We used acute kidney injury (AKI) within the first 48 hours (according to Kidney Disease Improving Global Outcomes (KDIGO) definition), ICU mortality and hospital mortality as outcome parameters. Multilevel logistic regression with the ICU unit as random effect and overdose versus all other diagnoses as fixed effect was used to calculate adjusted odds ratios (aOR) with respective 95% confidence intervals (95%CI). Additionally, we conducted multivariable adjustment for age, sex, SOFA score and ethnicity. We chose these covariables based on our clinical experience. In a sensitivity analysis (with the subgroups: sex, age categories, ethnicity (Caucasian vs. Non-Caucasian), teaching vs. non-teaching hospitals, maximum lactate day 1 (< or ≥2.0 mmol/l), mechanical ventilation and vasopressor use), hospital mortality of intoxicated versus all other ICU patients was compared. Stata/IC 16.1 (StataCorp. 2019. Stata Statistical Software: Release 16. College Station, TX: StataCorp LLC) was used for all statistical analyses.

## Results

### Characteristics of intoxicated versus other ICU patients

Regarding baseline characteristics, intoxicated patients were younger (median (IQR): 41 (29-53) yrs. vs. 66 (54-77) yrs.; *p*<0.001), as well as significantly more often female (55 vs. 45%; *p*<0.001). There was also a non-significant difference with regard to body mass indices (BMI) with more normal weighted patients in the “intoxication group” (36% vs. 30%) to more obese individuals in the other group (37 vs. 31%; *p*<0.001). More Caucasian (83 vs. 78%) and fewer African American (8 vs. 12%; *p*<0.001) patients were observed in the “intoxication group”. Intoxicated patients were also "healthier" in terms of disease severity according to common intensive care scores (sequential organ failure assessment/SOFA; and Acute Physiology and Chronic Health Evaluation/APACHE-IV), and laboratory parameters (blood counts, creatinine and lactate). The frequency of vasopressor use (5 vs. 15%; *p*<0.001) was lower, while rates of mechanical ventilation were higher (24 vs. 26%; *p*<0.001) in intoxicated subjects. There were no differences regarding dialysis in the first three days (3 vs. 4%; *p*=0.26). Accordingly, a significant difference for all outcome parameters was observed. A detailed list of all parameters is given in Table 1.

**Table 1:** Comparison of general characteristics, laboratory values, intensive care measures and outcome parameters between intoxicated patients and other ICU patients from the eICU database

Characteristic	Other diagnoses (***n***=105,998)	Intoxications (***n***=4,267)	***p***-value
Age (years) *– median (IQR)*	66 (54-77)	41 (29-53)	<0.001*
*Age category*	-	-	<0.001*
Age <65 years	47% (49,725)	92% (3,934)	-
Age 65-79 years	33% (35,431)	6% (246)	-
Age ≥80 years	20% (20,842)	2% (87)	-
*Sex*	-	-	<0.001*
Female	45% (48,213)	55% (2,333)	-
Male	54% (57,757)	45% (1,929)	-
Other	0% (7)	0% (0)	-
Unknown	0% (19)	0% (3)	-
BMI (kg/m^2^) *– median (IQR)*	28 (23-33)	26 (23-31)	<0.001*
*BMI categories (WHO)*	-	-	<0.001*
BMI <18.5 kg/m^2^	4% (4,617)	4% (160)	-
BMI 18.5 to <25 kg/m^2^	30% (30,747)	36% (1,468)	-
BMI 25 to <30 kg/m^2^	29% (30,027)	30% (1,224)	-
BMI ≥30 kg/m^2^	37% (38,416)	31% (1,261)	-
*Ethnicity*	-	-	<0.001*
African American	12% (12,399)	8% (324)	-
Asian	1% (1,491)	1% (45)	-
Caucasian	78% (81,375)	83% (3,462)	-
Hispanic	4% (4,133)	3% (112)	-
Native American	1% (693)	1% (54)	-
Other/Unknown	5% (4,762)	4% (162)	-
SOFA score (pts.) *– median (IQR)*	4 (2-6)	3 (1-5)	<0.001*
APACHE-IV (pts.) *– median (IQR)*	52 (38-70)	37 (26-54)	<0.001*
*Laboratory values – median (IQR)*	-	-	-
Maximum lactate day 1 (mmol/L)	1.9 (1.2-3.3)	1.6 (1.0-2.5)	<0.001*
First lactate >2 mmol/L	40% (12,699)	35% (324)	<0.001*
Maximum creatinine day 1 (mg/dL)	1.0 (0.8-1.6)	0.8 (0.7-1.0)	<0.001*
Hemoglobin (g/dL)	11.2 (9.5-12.8)	12.4 (11.3-13.6)	<0.001*
Platelets (G/L)	192.0 (144.0-249.0)	210.0 (169.0-256.0)	<0.001*
Leucocytes (G/L)	10.4 (7.5-14.5)	8.7 (6.5-11.6)	<0.001*
*Diagnosis category*	-	-	<0.001*
ACS	8% (8,343)	0% (0)	-
ARF	2% (1,932)	0% (0)	-
Asthma/Emphysema	4% (3,948)	0% (0)	-
CABG	5% (4,771)	0% (0)	-
CHF	5% (5,592)	0% (0)	-
CVA	9% (9,758)	0% (0)	-
CV (others)	3% (3,593)	0% (0)	-
Cardiac Arrest	9% (9,135)	0% (0)	-
Coma	2% (2,082)	0% (0)	-
DKA	4% (4,384)	0% (0)	-
GI bleeding	7% (7,277)	0% (0)	-
GI obstruction	1% (1,232)	0% (0)	-
Neurological	4% (4,640)	0% (0)	-
Overdose	0% (0)	100% (4,267)	-
Pneumonia	4% (4,577)	0% (0)	-
Respiratory (others)	8% (7,970)	0% (0)	-
Sepsis	17% (18,087)	0% (0)	-
Trauma	6% (5,884)	0% (0)	-
Valvular Disease	3% (2,793)	0% (0)	-
*Intensive care measures*	-	-	-
Mechanical ventilation	24% (25,280)	26% (1,118)	<0.001*
Vasopressor use	15% (15,994)	5% (197)	<0.001*
Dialysis (first three days)	4% (2,048)	3% (36)	0.26
*Outcome*	-	-	-
AKI	6% (4,745)	2% (49)	<0.001*
LOS (hours)	45 (24-82)	28 (18-46)	<0.001*
LOS >7 days	9% (9,521)	3% (111)	<0.001*
ICU mortality	6% (6,827)	0% (21)	<0.001*
Hospital mortality	10% (10,661)	1% (32)	<0.001*

*Abbreviations*: *ACS* Acute coronary syndrome, *AKI* Acute kidney injury, *APACHE IV* Acute Physiology And Chronic Health Evaluation, *ARF* Acute respiratory failure, *BMI* Body mass index, *CABG* Coronary artery bypass grafting, *CHF* Congestive heart failure, *CVA* Cerebrovascular accidents, *CV* Cerebrovascular, *DKA* Diabetic ketoacidosis, *GI* Gastro-intestinal, *ICU* Intensive care unit, *LOS* Length of stay, *SOFA* Sequential organ failure assessment, *WHO* World health organization; *: statistically significant

The odds for hospital mortality were lower in intoxicated patients (aOR 0.07 95%CI 0.05-0.11; *p*<0.001), a finding which persisted after multivariable adjustment for age, sex, ethnicity and SOFA score (aOR 0.17 95%CI 0.12-0.24; *p*<0.001).

### Sensitivity analysis

**Fig. 1:**
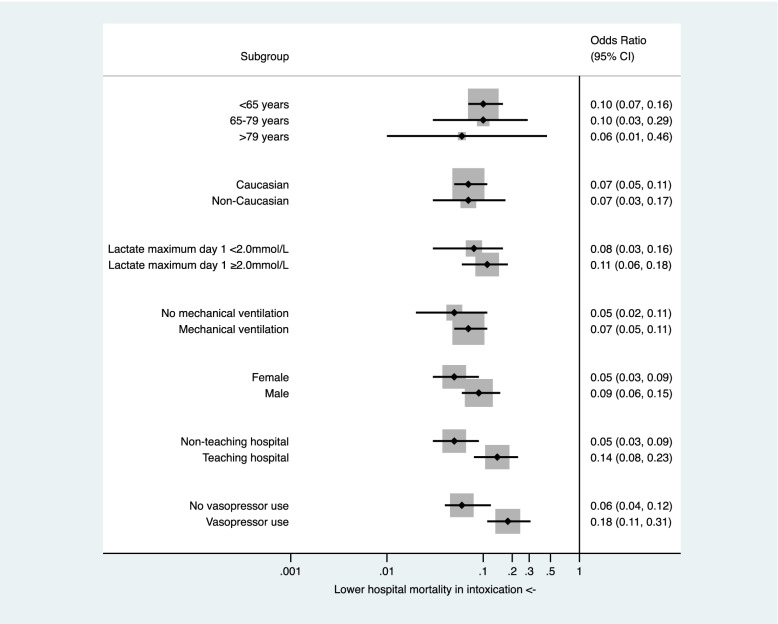
Sensitivity analysis of hospital mortality of intoxicated versus all other ICU patients by means of different subgroups (Forest plot). Abbreviations: aOR: adjusted odds ratio; CI: confidence interval; ICU: intensive care unit

This finding was confirmed for the entire subgroup analysis in terms of type of hospital (non-teaching OR 0.05 CI 0.03-0.09; teaching OR 0.14 CI 0.08-0.23), age (<65 years OR 0.10 CI 0.07-0.16; 65-79 years OR 0.10 CI 0.03-0.29; >79 years OR 0.06 CI 0.01-0.46), ethnicity (Caucasian OR 0.07 CI 0.05-0.11; non-Caucasian OR 0.07 CI 0. 03-0. 17), maximum lactate at day 1 (<2.0mmol/L OR 0.08 CI 0.03-0.16; ≥2.0mmol/L OR 0.11 CI 0.06-0.18), mechanical ventilation (no MV OR 0.05 CI 0.02-0.11; MV OR 0. 07 CI 0.05-0. 11), sex (female OR 0.05 CI 0.03-0.09; male OR 0.09 CI 0.06-0.15), and vasopressor use (no vasopressor use OR 0.06 CI 0.04-0.12; vasopressor use OR 0.18 CI 0.11-0.31). In Fig. 1, the results of the subgroup analysis regarding mortality in intoxicated versus all other ICU patients are shown by means of a forest plot.

### Comparison of survivors and non-survivors of intoxicated patients

Regarding baseline characteristics of survivors and non-survivors, there was a numerical but no statistically significant difference for age (median (IQR): 41 (29-53) years in survivors; 48 (30-60) years in non-survivors; *p*=0.32), as well as for sex (55% female patients in survivors vs. 41% in non-survivors; *p*=0.27). Non-survivors were more often under- or normally weighted and less often overweighted or obese according to the World Health Organization (WHO) classification (see Table 2). Disease severity assessed by intensive care scores was higher in non-survivors (median SOFA: 8 vs. 3 pts.; APACHE-IV: 90 vs. 37 pts.). Also, patients in this group showed higher initial lactate (median 3.1 vs. 1.6 mmol/L; *p*<0.001), and serum creatinine values (median 1.3 vs. 0.8 mg/dL; *p*<0.001), as well as significantly higher leucocyte counts (median 13.0 vs. 8.6 G/l; *p*<0.001). In survivors, alcohols were observed as causative substances more frequently (12 vs. 0%), whereas non-survivors more often took street drugs (e.g. opiates, cocaine and amphetamine; 34 vs. 20%). Regarding outcome measures, higher rates of AKI (9 vs. 2%, *p*=0.047) and longer ICU stays were observed in non-survivors (see Table 2).

**Table 2:** Comparison of general characteristics, laboratory values, intensive care measures and outcome parameters between survivors and non-survivors in intoxicated patients.

Characteristic	Survivors (***n***=4,235)	Non-Survivors (***n***=32)	***p***-value
Age (years) *– median (IQR)*	41 (29-53)	48 (30-60)	0.32
*Age category*	-	-	0.61
Age <65 years	92% (3,906)	88% (28)	-
Age 65-79 years	6% (243)	9% (3)	-
Age ≥80 years	2% (86)	3% (1)	-
*Sex*	-	-	0.27
Female	55% (2,320)	41% (13)	-
Male	45% (1,910)	59% (19)	-
Unknown	0% (3)	0% (0)	-
BMI (kg/m^2^) *– median (IQR)*	27 (23-31)	24 (21-30)	0.16
*BMI categories (WHO)*	-	-	0.030*
BMI <18.5 kg/m^2^	4% (156)	13% (4)	-
BMI 18.5-24.9 kg/m^2^	36% (1,455)	43% (13)	-
BMI 25-29.9 kg/m^2^	30% (1,218)	20% (6)	-
BMI ≥30 kg/m^2^	31% (1,254)	23% (7)	-
*Ethnicity*	-	-	0.93
African American	8% (322)	6% (2)	-
Asian	1% (45)	0% (0)	-
Caucasian	83% (3,435)	84% (27)	-
Hispanic	3% (111)	3% (1)	-
Native American	1% (53)	3% (1)	-
Other/Unknown	4% (161)	3% (1)	-
SOFA score (pts.) *– median (IQR)*	3 (1-5)	8 (5-11)	<0.001*
APACHE-IV (pts.) *– median (IQR)*	37 (26-53)	90 (64-113)	<0.001*
*Laboratory values – median (IQR)*	-	-	-
Maximum lactate day 1 (mmol/L)	1.6 (1.0-2.5)	3.1 (1.7-6.4)	<0.001*
First lactate >2mmol/L	34% (309)	71% (15)	<0.001*
Maximum creatinine day 1 (mg/dL)	0.8 (0.7-1.0)	1.3 (1.0-2.3)	<0.001*
Hemoglobin (g/dL)	12.4 (11.3-13.6)	12.6 (10.4-14.8)	0.68
Platelets (G/L)	210 (169-256)	224.5 (173.0-276.0)	0.40
Leucocytes (G/L)	8.6 (6.5-11.6)	13.0 (9.2-18.2)	<0.001*
*Toxin/poison/drug*	-	-	0.22
Overdose alcohols^(1)^	12% (521)	0% (0)	-
Overdose analgesics^(2)^	11% (480)	16% (5)	-
Overdose antidepressants^(3)^	7% (301)	6% (2)	-
Overdose of other toxin/poison/drug	18% (778)	19% (6)	-
Overdose sedatives^(4)^	29% (1,249)	25% (8)	-
Overdose, street drugs^(5)^	20% (852)	34% (11)	-
Drug-toxicity^(6)^	1% (54)	0% (0)	-
*Intensive care measures*	-	-	-
Mechanical ventilation	26% (1,097)	66% (21)	<0.001*
Vasopressor use	4% (184)	41% (13)	<0.001*
Dialysis (first three days)	3% (35)	6% (1)	0.50
*Outcome*	-	-	-
AKI	2% (47)	9% (2)	0.047*
LOS (hours)	28 (18-46)	76 (35-175)	<0.001*
LOS >7 days	2% (103)	25% (8)	<0.001*
ICU mortality	0% (0)	66% (21)	<0.001*
Hospital mortality	0% (0)	100% (32)	<0.001*

*Abbreviations*: *AKI* Acute kidney injury, *APACHE IV* Acute Physiology And Chronic Health Evaluation, *BMI* Body mass index, *ICU* Intensive care unit, *LOS* Length of stay, *SOFA* Sequential organ failure assessment; *WHO* World health organization; (1) ethanol, methanol, ethylene glycol; (2) aspirin, acetaminophen; (3) tricyclic antidepressants, lithium; (4) including hypnotics, antipsychotics & benzodiazepines; (5) opiates, cocaine, amphetamine; (6) i.e., beta blockers, calcium channel blockers, etc.; *: statistically significant;

### Comparison of different substance categories

As for causative substances, individuals with drug toxicity were significantly older (median age 70 vs. 37-44 years in all other groups; *p*<0.001), whereas for all other categories most patients were <65 years old (90-96% vs. 41%; *p*<0.001). In particular, analgesics, antidepressants, sedatives and drug toxicity were more frequently observed in women, and alcohol and street drugs were more common in men (see Table 3). Regarding BMI categories, a significant difference was found especially for alcohols with more normal weighted and less obese patients. Relevant differences were also found with regard to ethnicity. A detailed list of the specific differences can be found in Table 3. In terms of disease severity by means of APACHE-IV, the lowest scores were observed for analgesics (30 pts.), whereas the highest scores were found for drug toxicity (55 pts.; *p*<0.001). As for laboratory values, the highest lactate values on day 1 were found in alcohols (median 2.1 vs. 1.4-1.8 mmol/L; *p*<0.001), while the highest serum creatinine values were found in the drug toxicity group (median 1.1 vs. 0.8-0.9 mg/dL; *p*<0.001). The lowest rates of mechanical ventilation were observed in analgesics and drug toxicity (13 and 11% vs. 25-30% in all other groups; *p*<0.001), whereas patients with drug toxicity needed vasopressors significantly more often (17% vs. 2-6%; *p*<0.001). This group was also most likely to require dialysis within the first three days (13% vs. 2-6%; *p*=0.035). No significant differences were observed for outcome between AKI and mortality (see Table 3). Regarding length of stay, the shortest stays were found in the alcohols-group (median 24 hours), the longest in the drug toxicity-group (median 34 hours; *p*<0.001).

**Table 3:** Comparison of general characteristics, laboratory values, intensive care measures and outcome parameters between different substance categories in intoxicated patients.

	Alcohols^**(1)**^ (***n***=521)	Analgesics^**(2)**^ (***n***=485)	Antidepressants^**(3)**^ (***n***=303)	Others^**(4)**^ (***n***=784)	Sedatives^**(5)**^ (***n***=1,257)	Street drugs^**(6)**^ (***n***=863)	Drug toxicity^**(7)**^ (***n***=54)	***p***-value
Age (years) *– median (IQR)*	43 (32-52)	38 (25-53)	37 (27-49)	39 (28-52)	44 (33-54)	38 (28-51)	70 (49-83)	<0.001*
*Age category*	-	-	-	-	-	-	-	<0.001*
Age <65 years	96% (502)	90% (436)	96% (292)	92% (718)	92% (1,158)	93% (806)	41% (22)	-
Age 65-79 years	3% (16)	8% (41)	3% (9)	5% (43)	6% (81)	5% (42)	26% (14)	-
Age ≥80 years	1% (3)	2% (8)	1% (2)	3% (23)	1% (18)	2% (15)	33% (18)	-
*Sex*	-	-	-	-	-	-	-	<0.001*
Female	36% (187)	68% (329)	65% (196)	56% (441)	62% (775)	43% (368)	69% (37)	-
Male	64% (332)	32% (156)	35% (107)	44% (342)	38% (481)	57% (494)	31% (17)	-
Unknown	0% (1)	0% (0)	0% (0)	0% (0)	0% (1)	0% (1)	0% (0)	-
BMI (kg/m^2^) *– median (IQR)*	25 (22-29)	27 (23-32)	26 (23-32)	27 (23-32)	27 (23-32)	26 (23-31)	26 (23-30)	<0.001*
*BMI categories (WHO)*	-	-	-	-	-	-	-	<0.001*
BMI <18.5 kg/m^2^	5% (26)	6% (26)	4% (11)	4% (29)	4% (47)	2% (20)	2% (1)	-
BMI 18.5-<25 kg/m^2^	43% (216)	34% (160)	34% (99)	35% (263)	32% (392)	38% (318)	38% (20)	-
BMI 25-<30 kg/m^2^	32% (161)	28% (132)	30% (88)	29% (222)	28% (343)	31% (261)	32% (17)	-
BMI ≥30 kg/m^2^	20% (100)	31% (146)	32% (95)	33% (248)	35% (426)	28% (231)	28% (15)	-
*Ethnicity*	-	-	-	-	-	-	-	<0.001*
African American	6% (33)	8% (38)	7% (21)	10% (80)	5% (64)	10% (81)	13% (7)	-
Asian	1% (7)	2% (11)	0% (1)	1% (11)	1% (8)	1% (7)	0% (0)	-
Caucasian	81% (412)	79% (368)	87% (259)	80% (608)	88% (1,077)	82% (699)	74% (39)	-
Hispanic	4% (19)	3% (15)	1% (3)	3% (23)	2% (30)	2% (18)	8% (4)	-
Native American	2% (12)	2% (7)	1% (3)	2% (13)	1% (14)	1% (5)	0% (0)	-
Other/Unknown	5% (27)	6% (27)	3% (10)	4% (28)	2% (27)	5% (40)	6% (3)	-
SOFA-score (pts.) *– median (IQR)*	3 (1-4)	2 (1-4)	2 (1-4)	3 (1-5)	3 (1-5)	3 (2-5)	3 (2-5)	<0.001*
APACHE-IV (pts.) *– median (IQR)*	36 (26-50)	30 (22-45)	35 (25-49)	38 (27-56)	39 (28-55)	40 (28-55)	55 (38-70)	<0.001*
*Laboratory values – median (IQR)*	-	-	-	-	-	-	-	-
Maximum lactate day 1 (mmol/l)	2.1 (1.3-3.3)	1.4 (1.0-2.3)	1.4 (1.0-2.3)	1.8 (1.2-2.6)	1.6 (0.9-2.4)	1.4 (0.9-2.5)	1.2 (0.7-2.1)	<0.001*
First lactate >2mmol/L	51% (55)	24% (27)	27% (17)	40% (74)	32% (76)	32% (72)	27% (3)	<0.001*
Maximum creatinine day 1 (mg/dL)	0.8 (0.6-0.9)	0.8 (0.6-1.0)	0.8 (0.7-0.9)	0.8 (0.7-1.0)	0.8 (0.7-1.0)	0.9 (0.7-1.1)	1.1 (0.8-1.8)	<0.001*
Hemoglobin (g/dL)	12.7 (11.7-13.9)	12.3 (11.0-13.6)	12.4 (11.3-13.3)	12.5 (11.3-13.6)	12.4 (11.3-13.5)	12.4 (11.1-13.6)	11.4 (10.3-13.0)	<0.001*
Platelets G/L	194 (148-245)	225 (176-271)	214 (175-257)	214 (170-260)	209 (174-257)	207 (170-249)	218 (164-256)	<0.001*
Leucocytes (G/L)	7.5 (5.7-10.0)	8.4 (6.3-11.4)	8.7 (6.8-11.6)	8.8 (6.5-11.6)	8.5 (6.5-11.4)	9.5 (7.1-12.9)	9.5 (6.6-11.3)	<0.001*
*Intensive care measures*	-	-	-	-	-	-	-	-
Mechanical ventilation	27% (140)	13% (61)	25% (77)	25% (197)	30% (375)	30% (262)	11% (6)	<0.001*
Vasopressor use	2% (8)	3% (15)	5% (14)	6% (50)	5% (62)	5% (39)	17% (9)	<0.001*
Dialysis (first 3 days)	2% (3)	5% (5)	6% (5)	5% (10)	1% (5)	3% (6)	13% (2)	0.035*
*Outcome*	-	-	-	-	-	-	-	-
AKI	4% (8)	1% (3)	1% (2)	2% (6)	3% (16)	3% (13)	3% (1)	0.49
LOS (hours)	24 (15-41)	29 (19-43)	31 (19-45)	28 (17-48)	30 (18-48)	27 (17-47)	34 (23-49)	<0.001
LOS >7 days	2% (12)	2% (8)	4% (12)	3% (21)	3% (34)	3% (22)	4% (2)	0.61
ICU mortality	0% (0)	1% (3)	1% (2)	1% (6)	0% (6)	0% (4)	0% (0)	0.62
Hospital mortality	0% (0)	1% (5)	1% (2)	1% (6)	1% (8)	1% (11)	0% (0)	0.22

*Legend*: Comparison of general characteristics, laboratory values, intensive care measures and outcome parameters between different substance categories in intoxicated patients. *Abbreviations*: *AKI* Acute kidney injury; antidepr.: antidepressants, *APACHE IV* Acute Physiology And Chronic Health Evaluation, *BMI* Body mass index, *ICU* Intensive care unit, *LOS* Length of stay, *SOFA* Sequential organ failure assessment, *WHO* World health organization; (1) ethanol, methanol, ethylene glycol; (2) aspirin, acetaminophen; (3) tricyclic antidepressants, lithium; (4) other toxin/poison/drug; (5) including hypnotics, antipsychotics & benzodiazepines; (6) opiates, cocaine, amphetamine; (7) i.e., beta blockers, calcium channel blockers, etc.; *: statistically significant

## Discussion

Using a large cohort of over 100,000 ICU patients, this study demonstrates that intoxicated individuals have a better short-term outcome than patients with other critical illnesses regardless of initial lactate, as well as various baseline (age, sex, ethnicity), and patient care characteristics (level of care, mechanical ventilation, vasopressor use). Since intoxications can be caused by a vast variety of substances and poisoned patients present with a wide range of clinical manifestations, they have always been a challenge for acute care physicians. Despite the etiologic diversity and relatively high general prevalence of intoxicated patients in ICUs, the need for invasive measures is comparatively rare. This analysis is intended to provide further important data regarding specific patient characteristics of intoxicated patients, and in particular those individuals who have a poor outcome, in order to help to identify them early in terms of better resource allocation.

In general, we observed a significantly lower mortality of intoxicated patients compared to the entire remaining collective. It is certainly not without reason that “intoxication” is described in current resuscitation guidelines as "reversible cause" and usually represents an acute event with comprehensible pathophysiology and clinical course, frequently also without relevant pre-existing (chronic) organ damage [12]. With regard to outcome parameters, we found "less ill" patients in the intoxication group. Despite from similar rates of dialysis in the first three days, less patients suffered from AKI in the group of intoxicated patients. This is in accordance to our clinical experience, since the elimination of many substances can be accelerated with hemodialysis and/or –filtration [13]. A somewhat surprising finding is the higher proportion of women with acute poisoning, as a greater proportion of men in this patient population is frequently described in the literature [14]. A possible explanation could be intentional overdose (which is observed more frequently in women in contrast to e.g. exposure to chemicals) and high rates of sedatives as causative substances in our population which are used more often by female individuals [14]. However, Brandenburg et al. observed a similar finding in their large-scale analysis on intoxicated patients, as well as a likewise relatively young average age of patients [2]. Also, male patients had a numerically worse outcome compared to their female counterparts. This finding cannot be explained causally on the basis of the available data. Intoxicated patients in our study had lower BMIs, whereas underweighted patients within this group had worse outcomes. In the past a higher mortality in underweighted individuals has been observed in several medical conditions, as well as in the general population [15, 16]. In contrast, obese patients often show better outcomes, which is also known as the “obesity paradox”. The causes for weight-related outcome differences are complex and range from severe pre-existing chronic diseases, different drug distribution patterns of especially lipophilic substances, over nutritional status, socioeconomic factors up to immunological phenomenons [15–17]. For an accurate assessment of BMI-related outcomes, a standardized nutritional assessment, a detailed analysis of pre-existing conditions, but also a functional analysis regarding activities of daily life (ADLs) would be necessary. Unfortunately, we couldn’t obtain these data for this patient population, although the thesis-generating nature of the statement seems valuable. As for ethnicity, we observed a statistically significant difference between intoxicated and other ICU patients (more Caucasian patients in the “Intoxicated” group) as well as in-between groups of different causative substances (see Table 3), but not for mortality. In general, ethnic differences in drug overdose mortality have been observed in the US in the past [18]. Thus a lower mortality among Caucasian patients, but increasing mortality among African American and Hispanic individuals was observed, yet also co-involvement of other drugs varies with ethnicity [18]. Overall, for such an analysis, a distinction must be made between prescribed and illegal opioids, for example, and co-involvement of other substances has to be investigated. Yet we cannot provide a clear explanation for our findings as an analysis for causality is too complex and beyond the scope of this study. In general, age-group stratified, socioeconomic, educational and media-triggered factors must be considered as well [18]. We also found a marked difference for initial serum lactate in survivors compared to non-survivors. This finding has been described in the past, but the proportion of deceased patients in our cohort is too small to calculate optimal cut-offs and risk groups [19]. Especially in the group of patients intoxicated with alcohols, a high initial lactate was observed. This can be explained by an altered mitochondrial metabolism with reduced utilization in both acute and chronic alcoholism [20]. However, this group showed the best outcome, which highlights the importance of initial serum lactate in the other groups. With regard to further laboratory parameters, relevant differences were shown for all blood count parameters. The lowest hemoglobin values were found in the drug toxicity group, as well as a higher median serum creatinine. A possible explanation could be the significantly higher age of these patients. The lowest platelet counts were found in the “alcohols group”. Harmful alcohol consumption is often associated with qualitative and quantitative disturbances of platelet integrity [21]. Another interesting finding is that in two groups (“drug toxicity” and “street drugs”) significantly higher leukocyte counts were observed. In general, the frequent occurrence of idiopathic leukocytosis after use of stimulant drugs (so called “uppers”) has been described in the past, especially for amphetamines [22]. With regard to higher leukocyte counts in the “drug toxicity” group, a causal explanation is again not possible in the absence of precise data regarding the causative substances. In general, a variety of drugs can cause leukocytosis, whereas a sole delimitation of infectious causes by leukocytosis is not possible in the absence of other laboratory parameters [23]. It is also interesting to note that patients with “drug toxicity” had the highest proportion of vasopressor use and dialysis in the first three days, but a zero percent short term mortality. In contrast, significantly more mechanically ventilated patients were found in the "sedatives" and "street drugs" subgroups, with these individuals again contributing the highest numerical proportion of non-survivors. This possibly underlines the need for reversibility of intoxication, as mechanical ventilation per se is a known and relatively invasive ICU measure and independent predictor of ICU mortality [24].

## Conclusion

This large-scale retrospective analysis shows a significantly better outcome of intoxicated individuals compared to general ICU patients. In general, a very low mortality rate is observed in this patient collective. Yet, it is difficult to find the right balance between a sufficiently cautious approach regarding monitoring and safe outpatient- or low-level care in clinical routine. Risk stratification tools and scores are absolutely necessary to enable sufficient resource allocation in the future. Therefore, clearly structured and coherent data acquisition is essential. Further studies should generally focus on pre-existing functional and nutritional status, as well as its causes in and effects on the critically ill, but also intoxicated patients.

### Strengths and limitations

The great strength of this study is certainly the large number of included patients and the real-world character of the data. The main limitation of this study is its purely retrospective and observational character and the comparatively low number of deceased patients. This also prevents the use of an exact matching process. Also, the study only involves U.S. patients - in other countries, the results could be different. Since the eICU database uses the admission diagnoses according to APACHE IV, some patients may have been misclassified (e.g. "coma of unknown origin"). Due to the retrospective nature of the study, this issue cannot be excluded. Unfortunately, we do not have information on the location where the poisoning occurred (household, workplace, etc.), which might allow a better understanding of some data. Also, a more precise characterization of non-survivors (Invasiveness of measures, duration of ventilation, etc.) would be desirable. Nevertheless, we think that this large-scale study on more than 4,000 intoxicated individuals is a relevant contribution to the outcome assessment of intoxicated patients and can be thesis-generating for future studies.

## Data Availability

The data supporting the findings of this study are freely available at https://eicu-crd.mit.edu/. These data are publicly available online (after registration and signing a data use agreement). All other data in this particular manuscript are available from the corresponding author (Prof. Christian Jung) upon reasonable request.

## Abbreviations

ACS: Acute coronary syndrome
ADL: Activities of daily life
AKI: Acute kidney injury
APACHE: Acute Physiology And Chronic Health Evaluation
ARF: Acute respiratory failure
BMI: Body mass index
CABG: Coronary artery bypass grafting
CI: Confidence interval
CHF: Congestive heart failure
CVA: Cerebrovascular accidents
CV: Cerebrovascular
DKA: Diabetic ketoacidosis
GI: Gastro-intestinal
ICU: Intensive Care Unit
IQR: Interquartile range
LOS: Length of stay
MV: Mechanical ventilation
OR: Odds ratio
SOFA: Sequential Organ Failure Assessment
VP: Vasopressor
WHO: World health organization
